# Homocysteine is a bystander for ST-segment elevation myocardial infarction: a case-control study

**DOI:** 10.1186/s12872-018-0774-8

**Published:** 2018-02-13

**Authors:** Ching-Yu Julius Chen, Tzu-Ching Yang, Christopher Chang, Shao-Chun Lu, Po-Yuan Chang

**Affiliations:** 1 Cardiovascular Center and Division of Cardiology, Department of Internal Medicine, National Taiwan University Hospital, 7 Chung-Shan South Road, 100 Taipei, Taiwan; 2 Division of Cardiology, Department of Internal Medicine, National Taiwan University College of Medicine, No.1, Ren-Ai Road Section 1, 100 Taipei, Taiwan; 3 Department of Biochemistry and Molecular Biology, National Taiwan University College of Medicine, No.1, Ren-Ai Road Section 1, 100 Taipei, Taiwan; 4 Taipei American School, 800 Chung Shan North Road Section 6, Taipei, 11152 Taiwan

**Keywords:** Coronary artery disease, C-reactive protein (CRP), Homocysteine, ST-segment elevation myocardial infarction (STEMI), White blood cell (WBC)

## Abstract

**Background:**

Homocysteine has been long considered a risk factor for atherosclerosis. However, cardiovascular events cannot be reduced through homocysteine lowering by B vitamin supplements. Although several association studies have reported an elevation of serum homocysteine levels in cardiovascular diseases, the relationship of homocysteine with ST-segment elevation myocardial infarction (STEMI) is not well established.

**Methods:**

We prospectively enrolled STEMI patients who were consecutively admitted to an intensive care unit following coronary intervention in a single medical center in Taiwan. Control subjects were individuals who presented to the outpatient or emergency department with acute chest pain but subsequently revealed patent coronary arteries by coronary arteriography. The association between serum homocysteine levels and STEMI was investigated. A culture system using human coronary artery endothelial cells was also established to examine the toxic effects of homocysteine at the cellular level.

**Results:**

Patients with chest pain were divided into two groups. The STEMI group included 56 patients who underwent a primary percutaneous coronary intervention. The control group included 17 subjects with patent coronary arteries. There was no difference in serum homocysteine levels (8.4 ± 2.2 vs. 7.6 ± 1.9 μmol/L, *p* = 0.142). When stratifying STEMI patients by the Killip classification into higher (Killip III-IV) and lower (Killip I-II) grades, CRP (3.3 ± 4.1 vs. 1.4 ± 2.3 mg/L, *p* = 0.032), peak creatine kinase (3796 ± 2163 vs. 2305 ± 1822 IU/L, *p* = 0.023), and SYNTAX scores (20.4 ± 11.1 vs. 14.8 ± 7.6, *p* = 0.033) were significantly higher in the higher grades, while serum homocysteine levels were similar. Homocysteine was not correlated with WBCs, CRP, or the SYNTAX score in STEMI patients. In a culture system, homocysteine at even a supraphysiological level of 100 μmol/L did not reduce the cell viability of human coronary artery endothelial cells.

**Conclusions:**

Homocysteine was not elevated in STEMI patients regardless of Killip severity, suggesting that homocysteine is a bystander instead of a causative factor of STEMI. Our study therefore supports the current notion that homocysteine-lowering strategies are not essential in preventing cardiovascular disease.

## Background

Homocysteine is a highly reactive, sulfur-containing amino acid formed as a byproduct of the metabolism of the essential amino acid methionine [1], and methylenetetrahydrofolatereductase (MTHFR) is a key enzyme in this process. The serum level of homocysteine is significantly elevated in areas with dietary folate and B vitamin deficiencies as well as in subjects with the MTHFR 677 TT genotype [2], which can be lowered by B vitamins, including folic acid, B6, and B12. Homocysteine used to be considered a risk factor for atherosclerosis, which was primarily observed in children with extremely elevated serum homocysteine levels as well as premature atherothrombotic disease, and basic studies demonstrated that homocysteine can induce vascular damage by promoting platelet activation, oxidative stress, endothelial dysfunction, hypercoagulability, vascular smooth muscle cell proliferation, and endoplasmic reticulum stress [1, 3, 4]. A meta-analysis that collected large numbers of prospective studies and corrected for a regression dilution bias did show a significant association between the serum level and the incidence of cardiovascular disease (CVD) [5–10]. Serum levels were also correlated with long-term outcomes in patients with documented coronary artery disease (CAD) or acute coronary syndrome [11, 12]. However, recent studies disclosed that even though serum homocysteine levels were reduced by B vitamins in patients with stable CAD, acute coronary syndrome, or stroke, the rate of major adverse cardiac events was not reduced [13–17]. Moreover, there was no difference in early and late cardiovascular mortality among the tertiles of serum homocysteine levels in patients with ST-segment elevation myocardial infarction (STEMI) or non-ST-segment elevation acute coronary syndrome [18]. A possible explanation is the generally accepted concept of primary percutaneous coronary intervention (PCI) in STEMI, the common use of a new generation of drug-eluting stents in this era, [19, 20] and the development of a newer P2Y12 inhibitor, [21, 22] which has contributed to great improvements in survival and stent patency, thereby attenuating any potential benefits of homocysteine-lowering interventions. We tried to clarify the role of homocysteine in STEMI, an extreme entity of acute coronary syndrome, by making comparisons with subjects presenting with chest pain but proven to have patent or non-significant coronary arteries.

## Methods

### Study population

This study was approved by the institutional review board and conformed with the principles outlined in the *Declaration of Helsinki*. We prospectively enrolled STEMI patients who were consecutively admitted to an intensive care unit following coronary intervention in this study. The control group included individuals who presented to the outpatient or emergency department with acute chest pain but subsequently revealed patent coronary arteries by coronary arteriography. This study was carried out in a single medical center in Taiwan from March to September in 2014. STEMI was defined according to the 2013 American Heart Association (AHA)/American College of Cardiology (ACC) and European Society of Cardiology (ESC) guidelines, including persistent electrocardiographic (ECG) ST elevation and subsequent release of biomarkers of myocardial necrosis [23, 24]. All participants provided written informed consent.

### Study protocol

We recorded the time elapsed from exhibition of symptoms to receipt of medical services and the door-to-balloon time; demographic data and atherosclerotic risk factors including hypertension, diabetes, dyslipidemia, cigarette smoking, a family history of premature myocardial infarction, peripheral arterial disease, stroke, and the body-mass index; serum homocysteine, complete lipid profiles, hemoglobin A1c, white cell counts, C-reactive protein (CRP), peak creatine kinase (CK), and the time elapsed to the peak value; the severity of STEMI by the Killip classification and coronary anatomy by the SYNTAX score; life-threatening conditions including ventricular tachyarrhythmias, cardiac arrest, the use of an intra-aortic balloon pump (IABP) or extracorporeal membranous oxygenation (ECMO); echocardiographic parameters including the left ventricular ejection fraction (LVEF) and E/E’; electrocardiographic parameters including the PR and QTc interval, QRS duration, existence of fragmented QRS or early repolarization; and medications for acute coronary syndrome including antiplatelets, glycoprotein IIb/IIIa inhibitor, beta-blockade, angiotensin-converting enzyme inhibitor (ACEi) or angiotensin-II receptor blocker, statins, or antiarrhythmic drugs. We compared differences between the STEMI and control groups and analyzed whether there was a correlation between the severity of STEMI and the abovementioned parameters. All live STEMI patients were followed up for at least 1 month to evaluate their short-term prognosis.

### Cell cultures

Human coronary artery endothelial cells (ECs; HCAECs, Clonetics, US), at passages 4 to 7, were maintained in EGM-MV medium supplemented with 20% fetal bovine serum (FBS) and antibiotics. For the experiments, all cultures of subconfluent HCAECs were incubated with phosphate-buffered saline (PBS, as a control), homocysteine, cysteine, or electronegative L5 low-density lipoprotein (LDL) isolated from STEMI patients according to a previously described protocol [25, 26].

Cell viability 3-(4,5-dimethylthiazol-2-yl)-2,5-diphenyltetrazolium bromide (MTT) assay.

The chemical MTT was purchased from Sigma (St. Louis, MO) [26, 27]. HCAECs (5 × 10^4^ cells/well) were dispensed into 24-well plates and incubated for 24 h after the addition of homocysteine or different treatments, and the index of EC viability was determined by the colorimetric MTT (tetrazolium) assay. The absorbance was measured at a wavelength of 540 nm for viable cells using a microplate reader (Thermo Electron, Waltham, MA).

### Statistical analyses

A Chi-squared or Fisher exact test was used to compare categorical variables. Continuous variables were determined by either Student’s *t*-test or a one-way analysis of variance (ANOVA). All continuous data are expressed as means ± standard deviation. A two-tailed *p* value of < 0.05 was considered statistically significant. Regression analysis with a linear model was used to analyze the correlation between continuous parameters. Statistical analysis was performed using IBM SPSS Statistics for Windows, Version 22.0 (Armonk, NY).

## Results

We enrolled a total of 73 patients with chest pain. The STEMI group included 56 patients who underwent a primary PCI for total coronary occlusion. The control group included 17 subjects who presented with chest pain but had patent coronary arteries. In the control group, two patients visited the emergency department due to aggravating typical angina, 14 patients had stress-induced ischemia in thallium myocardial perfusion imaging, and one patient had progressive chest pain despite optimal medical treatment for reflux esophagitis; 11 patients were diagnosed as syndrome X, two patients had myocardial bridge, one patient had apical hypertrophy, one patient had coronary spasm, one patient had reflux esophagitis, and one patient had atrial fibrillation with rapid ventricular response. The demographic profile of study subjects is shown in Table 1. There was no difference in age, gender, or atherosclerosis risk factors such as hypertension, diabetes, dyslipidemia, and smoking between the two groups, as well as no differences in serum LDL and hemoglobin A1c. Two patients in the STEMI group died from cardiogenic shock; one had diabetes with multivessel coronary lesions. Among the 54 STEMI survivals, 12 were diabetes; 5 out of these 12 diabetic STEMI patients had multivessel lesions. None of our study subjects was on regular B-vitamin supplements for more than 1 year before enrollment.

**Table 1:** Demographic data of the ST-segment elevation myocardial infarction (STEMI) and control groups

	STEMI	Control	*p* value
Number, *n*	56	17	NA
Age, yr	58.1 ± 13	57.5 ± 11	0.854
Male, *n*(%)	43(77)	11(65)	0.353
Hypertension, *n*(%)	25(45)	9(53)	0.589
Diabetes, *n*(%)	13(23)	3(18)	0.748
Dyslipidemia, *n*(%)	23(41)	8(47)	0.781
Smoking, *n*(%)	33(59)	6(35)	0.103
Stroke, *n*(%)	7(13)	1(6)	0.672
Peripheral arterial disease, *n*(%)	0(0)	0(0)	NA
End-stage renal disease, *n*(%)	1(2)	0(0)	1.000
History of congestive heart failure, *n*(%)	1(2)	1(6)	0.414
Family history of myocardial infarction, *n*(%)	7(13)	2(12)	1.000
Body-mass index, kg/m^2^	25.1 ± 4	25.5 ± 6	0.835
Mortality, *n*(%)	2(4)	0(0)	1.000
Regular B-vitamin supplements> 1 year, *n*(%)	0(0)	0(0)	NA

*NA* not available

White blood cell (WBC) counts (11.9 ± 4 × 10^9^ vs. 6.5 ± 2 × 10^9^/L, *p* < 0.001), CRP (2.0 ± 3.0 vs. 0.2 ± 0.1 mg/L, *p* < 0.001), serum creatinine levels (1.2 ± 0.8 vs. 0.9 ± 0.2 mg/dL, *p* = 0.004), and QTc (441.1 ± 48 vs. 416.6 ± 21 ms, *p* = 0.044) were higher in STEMI patients. The LVEF was lower in the STEMI group (54.1% ± 12% vs. 69.6% ± 6%, *p* < 0.001) (Table 2). There was no significant difference in serum homocysteine levels (8.4 ± 2.2 vs. 7.6 ± 1.9 μmol/L, *p* = 0.142) after two outliers were excluded (Table 2). The two outliers (homocysteine of 36.8 and 32.3 μmol/L) were classified as Killip I and II, respectively, without renal impairment. The average follow-up time was 19.3 ± 1.9 months in the STEMI group, while one of the STEMI patients died of refractory ventricular fibrillation 4 days after the event day and another of pump failure 5 days later (Table 3). There was no difference in E/E’ values in echocardiographic measurements or the percentage of early repolarization shown in electrocardiograms between the two groups, while there was a trend of greater QRS fragmentation in the STEMI group (80% vs. 53%, *p* = 0.055).

**Table 2:** Comparison of laboratory, electrocardiographic, and echocardiographic parameters between the ST-segment elevation myocardial infarction (STEMI) and control groups

	STEMI	Control	*p* value
White cell count, ×10^9^/L	11.9 ± 4	6.5 ± 2	< 0.001*
Homocysteine, μmol/L	8.4 ± 2.2	7.6 ± 1.9	0.142^a^
C-reactive protein, mg/L	2.0 ± 3.0	0.2 ± 0.1	< 0.001*
Low-density lipoprotein, mg/dL	111.5 ± 38	95 ± 27	0.062
Creatinine, mg/dL	1.2 ± 0.8	0.9 ± 0.2	0.004*
Hemoglobin A1c, %	6.5 ± 1.6	6.1 ± 1.6	0.441
PR, ms	171.1 ± 43	173.6 ± 48	0.853
QRS, ms	94.9 ± 24	90.1 ± 12	0.273
QTc, ms	441.1 ± 48	416.6 ± 21	0.044*
QRS fragmentation, *n*(%)	45(80)	9(53)	0.055
Early repolarization, *n*(%)	18(32)	3(18)	0.362
Left ventricular ejection fraction, %	54.1 ± 12	69.6 ± 6	< 0.001*
E/E’	12.4 ± 5	12.4 ± 7	0.989

*p < 0.05

^a^After the two outliers of 36.8 and 32.3 μmol/L were excluded

**Table 3:** Relationship between the severity of ST-segment elevation myocardial infarction (STEMI) and laboratory parameters

	Killip I	Killip II	Killip III	Killip IV	*p* value
Number, *n*	35	5	2	14	
Age, years	60.0 ± 13.5	63.6 ± 13.5	57.5 ± 12.0	51.3 ± 10.6	0.141
Symptom to hospital, h	4.6 ± 5.1	1.2 ± 1.3	0.5 ± 0.7	5.6 ± 5.4	0.249
Aborted sudden death, *n*(%)	1(3)	0	0	6(43)	0.001*
VT/Vf, *n*(%)	1(3)	0	0	5(36)	0.007*
ECMO, *n*(%)	0	0	0	3(21)	0.023*
IABP, *n*(%)	0	0	0	9(64)	< 0.001*
Follow-up time, months	19.3 ± 2.1	18.0 ± 1.1	19.0 ± 1.8	16.9 ± 7.3	0.299
Mortality, *n*(%)	0	0	0	2(15)	0.082
White cell count, × 10^9^/L	11.44 ± 3.5	13.7 ± 1.9	13.7 ± 6.2	12.0 ± 4.9	0.577
	11.7 ± 3.4		12.2 ± 4.9		0.704
Homocysteine, μmol/L	9.2 ± 5.3	12.9 ± 10.9	11.0 ± 3.7	8.0 ± 2.4	0.361
	9.7 ± 6.1		8.4 ± 2.6		0.256
C-reactive protein, mg/L	1.4 ± 2.3	1.4 ± 2.5	3.6 ± 3.9	3.3 ± 4.3	0.208
	1.4 ± 2.3		3.3 ± 4.1		0.032*
Low-density lipoprotein Cholesterol, mg/dL	116 ± 38	102 ± 30	127 ± 49	101 ± 41	0.574
	114 ± 37		104 ± 41		0.434
Peak creatine kinase (CK), IU/L	2100 ± 1391	3737 ± 3604	2595 ± 2773	3967 ± 2134	0.016*
	2305 ± 1822		3796 ± 2163		0.023*
Time to peak CK, h	10.4 ± 4.6	9.8 ± 4.0	6.5 ± 5.0	8.6 ± 4.0	0.460
SYNTAX score	14.2 ± 7.6	19.0 ± 6.8	20.0 ± 7.1	20.46 ± 11.7	0.123
	14.8 ± 7.6		20.4 ± 11.1		0.033*

*ECMO* extracorporeal membranous oxygenation, *IABP* Intra-aortic balloon pump

*p < 0.05

When stratifying STEMI patients by the Killip classification, CRP (3.3 ± 4.1 vs. 1.4 ± 2.3 mg/L, *p* = 0.032), peak CK (3796 ± 2163 vs. 2305 ± 1822 IU/L, *p* = 0.023), and SYNTAX scores (20.4 ± 11.1 vs. 14.8 ± 7.6, *p* = 0.033) were higher in the group with increased severity (Table 3). Serum homocysteine levels did not increase with the severity of myocardial infarction (Table 3). There was also no difference in age, time from exhibition of symptoms to hospitalization, or time elapsed to peak CK (Table 3).

**Fig. 1:**
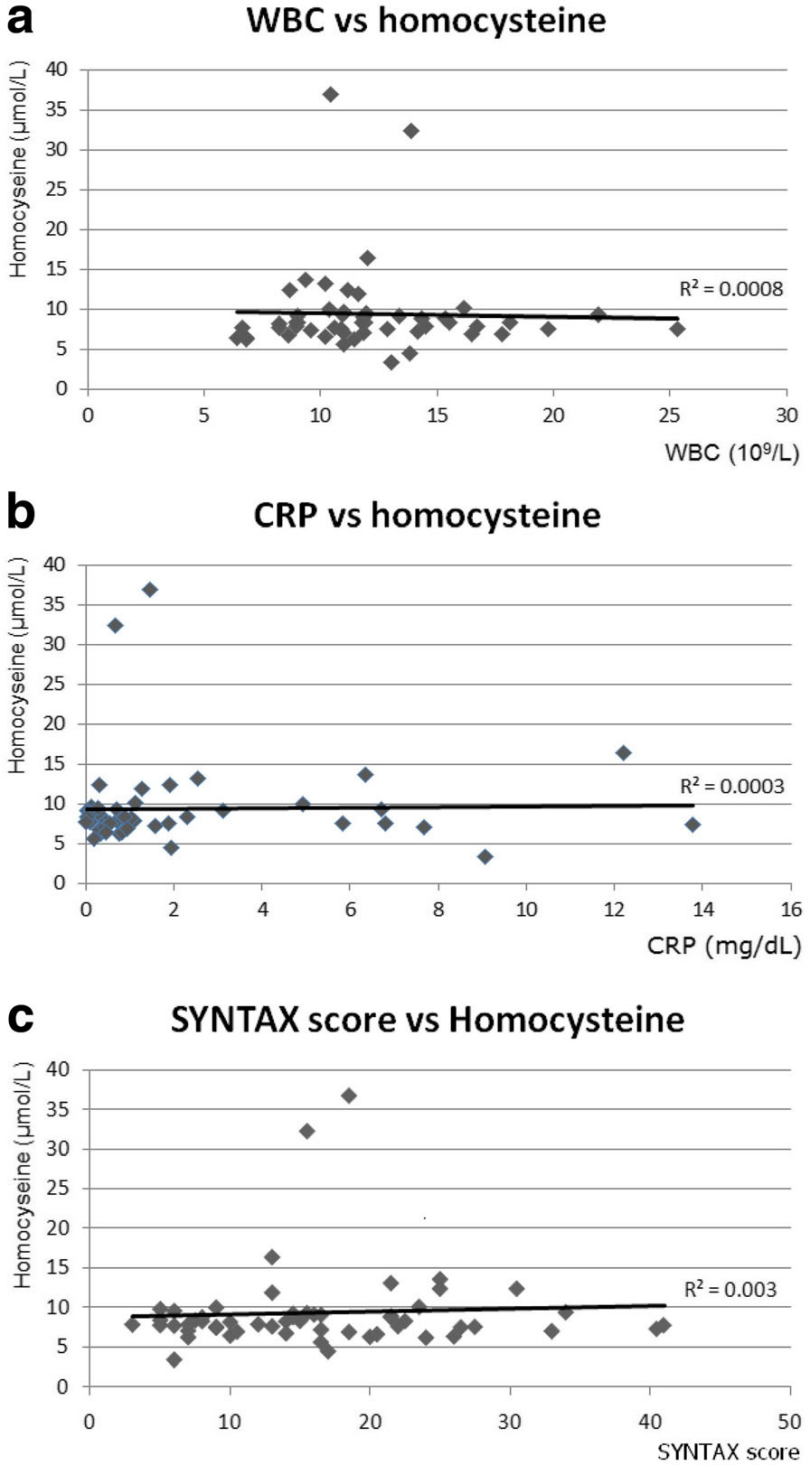
Relationship between homocysteine and three independent variables in ST-elevation myocardial infarction (STEMI) patients. **a** White blood cells (WBCs); **b** C-reactive protein (CRP); **c** SYNTAX score

When serum homocysteine levels were compared to various factors, we found that there was no correlation with WBC, CRP, or the SYNTAX score (Fig. 1a-c). In addition, the level of serum homocysteine was nearly consistent despite increases in these factors (*R*^2^ = 0.0008, 0.0003, and 0.003, respectively).

**Fig. 2:**
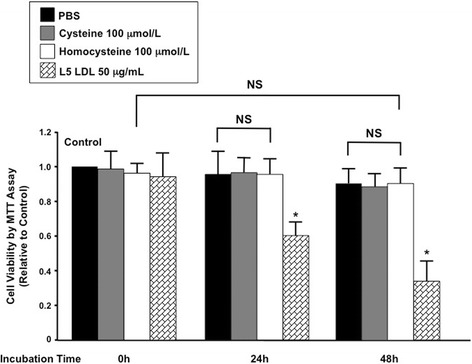
Effects of homocysteine on cell viability in cultured human coronary artery endothelial cells (HCAECs). Cells were treated with the PBS control, 100 μmol/L cysteine, 100 μmol/L homocysteine, or 50 μg/mL L5 low-density lipoprotein (LDL) for 0, 24, and 48 h as indicated, and cell viability was assessed by an MTT assay. Values are the mean±SEM (*n* = 3). * *p* < 0.05 vs. the PBS control (the first black column). NS, not significant

The clinical bystander role of homocysteine was further confirmed at the cellular level. A culture system was established using HCAECs and an MTT assay to assess cell viability in the presence of homocysteine (Fig. 2). Exposure of HCAECs to 100 μmol/L homocysteine for up to 48 h did not decrease cell viability compared with the PBS control (Fig. 2). In contrast, treatment with L5 LDL, an electronegative molecule known as a risk marker in STEMI, resulted in a time-dependent decrease in cell viability in HCAECs. Even at concentrations of > 100 μmol/L, homocysteine still did not change cell viability (data not shown). This bench evidence along with clinical observations in STEMI suggests that homocysteine is a bystander and not a causative factor of atherogenesis.

## Discussion

Our study clearly demonstrated that homocysteine was not elevated in STEMI patients, and therefore, this long-considered risk factor for CAD was more likely a bystander rather than a causative agent in STEMI. We believe our data are trustworthy because every study subject was evaluated in detail by coronary angiography. Our control group consisted of patients with the final diagnosis of Syndrome X, myocardial bridge [28], apical hypertrophy, coronary spasm, reflux esophagitis and atrial fibrillation, whose coronary patency was confirmed by coronary arteriography. This grouping contrasts with that reported in other studies in which “healthy” controls were based solely on non-invasive studies.

Although recent studies did not support the role of homocysteine as a risk factor in CVDs as previously thought, some reports have revealed an increase in homocysteine levels in CAD. A meta-analysis in 2002 reported that the mean serum homocysteine level in healthy populations at an average age of 56 years old was 11.8 μmol/L, and the odds ratio for ischemic heart disease associated with a 25% lower baseline homocysteine level was 0.83 (95% confidence interval (CI), 0.77~ 0.89) in prospective studies [8]. Akyurek et al. disclosed a higher serum homocysteine level in a STEMI group than in a healthy control group (19.0 ± 3.6 vs. 15.8 ± 4.2 μmol/L, *p* = 0.008) [10]. Liu C et al. found that the prevalence of hyperhomocysteinemia (> 15 μmol/L) was higher among patients with ischemic heart disease than among controls (79.1% vs. 5%) [29], and the serum homocysteine level was positively correlated with severity (acute myocardial infarction 23.44 ± 5.78 μmol/L, unstable angina 22.62 ± 6.37 μmol/L, stable angina 18.63 ± 6.73 μmol/L, and control 10.81 ± 4.62 μmol/L, *p <* 0.001) [30]. In contrast, our study showed that there was no difference in serum homocysteine levels between the control and STEMI groups, and our cohort had a mean level of homocysteine markedly lower than the levels reported in previous studies. Several public health studies have also shown a great diversity among serum homocysteine levels in different countries, ranging from 6.57 μmol/L in Kuwait [31] to 14 μmol/L in Italy [32], and folic acid fortification of grain products has already decreased the prevalence of high homocysteine levels (> 13 μmol/L) from 29.8% to 18.7% [33]. The variation in homocysteine levels may be attributed to ethnicity, socioeconomics, and nutritional status. Moreover, several studies demonstrating a neutral effect of homocysteine-lowering therapy in acute coronary syndrome have reported mean serum homocysteine levels of approximately 10 μmol/L [16, 34], relatively lower than those of previous studies, implying that the role of homocysteine in atherothrombotic heart disease may vary among populations with different average homocysteine levels. A trend of decreasing homocysteine levels in the general population also complicates the interaction of homocysteine and other established risk factors such as diabetes and smoking.

A previous study addressing the relationship of serum homocysteine levels with outcomes in patients with acute coronary syndrome showed that an elevated level on admission strongly predicted late cardiac events [12]. However, the rate of revascularization was 26.4% in that study, with an event rate of 9.3% over 28 days, which greatly differed from the current situation of acute coronary occlusion rapidly being treated through a percutaneous intervention. Because PCI has been demonstrated to be an indispensable strategy in acute coronary syndrome, the results of the abovementioned study with low revascularization rate cannot be applied to the era of aggressive and effective revascularization. Together with the findings gathered in our study, this result implies that advances in primary PCI in STEMI and the application of new-generation drug-eluting stents as well as newer P2Y12 inhibitors may attenuate the effect of serum homocysteine on cardiovascular outcomes.

One interesting finding of our study was the elevated leukocyte count in STEMI patients. It is well known that WBC counts are elevated in subjects with acute coronary syndrome [35], and leukocytosis is a predictor of major adverse cardiac events in patients with acute coronary syndrome [36, 37]. CRP is also a risk factor for cardiovascular events with a risk ratio of 1.67 (95% CI: 1.21~ 6.41) according to a meta-analysis of 42 prospective studies, [38] and it is an independent predictor of 30-day mortality in STEMI patients [39]. In our cohort, we observed significant increases in WBC and CRP levels in STEMI patients compared with the control group, and STEMI patients at higher Killip classes (III and IV) also had higher serum levels of these markers. However, the serum homocysteine level was consistently low regardless of the Killip class and had no correlation with WBC or CRP levels in STEMI patients. The discrepancy between our results and homocysteine’s role as a cardiovascular risk factor reported in previous studies requires a thorough reevaluation of homocysteine in the development of coronary heart disease and its prognostic value in patients with myocardial infarction.

STEMI patients had higher hemoglobin A1c levels than the control group in this prospective study, although the difference was not statistically significant. Diabetes has been known as a critical predictor of STEMI outcome. Indeed, our study showed that the diabetes% in the STEMI mortality subgroup (50%, 1 diabetes out of 2 mortality) was much higher than that in the STEMI survivals (21%, 12 diabetes out of 54 survivals). Recent studies showed that hyperglycemic stress during acute myocardial infarction (AMI) had a negative prognostic effect, [40] and diabetic patients with incretin-based therapy had a significant lower rate of all-cause mortality, cardiac death and readmission at 12 months [41]. Moreover, hyperglycemic patients with STEMI had fewer endothelial progenitor cells (EPCs) than normoglycemic patients, and an intensive glycemic control (80–140 mg/dL) group had a higher EPC number and the ability to differentiate and proportion salvaged myocardium, measured by technetium-99 m sestamibi scintigraphy [42]. These studies demonstrated that metabolic factors may directly affect the outcome of AMI and the viability of EPCs, and the latter may be a proxy for the probability of myocardial salvage.

Our in vitro experiments confirmed the bystander role of homocysteine in coronary artery cell damage (Fig. 2). As a long-considered risk factor for atherosclerosis, homocysteine can exhibit synergistic EC toxicity with modified LDL by a shared pathway related to fibroblast growth factor (FGF)-2 [26]. However, the homocysteine concentrations used in most experiments have been much higher than the physiological level, which is < 10 μmol/L, even under conditions with impaired folate metabolism [2]. In our study, under a supraphysiological concentration of 100 μmol/L, homocysteine still had no detrimental effects on HCAEC viability compared with its benign structural analog, cysteine. These results indicate that homocysteine is not the culprit molecule but instead a bystander in causing endothelial injury during cardiovascular events that arise from acute plaque rupture but not de novo thrombosis. Our findings have complicated the existing notions of the relationship between homocysteine and thrombosis [43] and may explain why homocysteine-lowering therapy has failed to reduce the risk of cardiovascular events. These observations offer a potential explanation for the negative results obtained from most clinical outcome trials on homocysteine reduction and raise a new topic of future homocysteine research.

## Conclusions

Unlike the leukocyte count and CRP, homocysteine was not elevated in STEMI patients regardless of Killip severity, suggesting that homocysteine is a bystander instead of a causative factor of STEMI. Our study therefore supports the current notion that homocysteine-lowering strategies are not essential in CVD prevention. Larger prospective studies are warranted to reevaluate the role of homocysteine in CAD.

## Abbreviations

CAD: Coronary artery disease
CRP: C-reactive protein
CVD: Cardiovascular disease
ECMO: Extracorporeal membranous oxygenation
IABP: Intra-aortic balloon pump
LDL: Low-density lipoprotein
LVEF: Left ventricular ejection fraction
PCI: Percutaneous coronary intervention
STEMI: ST-segment elevation myocardial infarction
WBC: White blood cell
